# Parechovirus A Infection of the Intestinal Epithelium: Differences Between Genotypes A1 and A3

**DOI:** 10.3389/fcimb.2021.740662

**Published:** 2021-11-01

**Authors:** Inés García-Rodríguez, Hetty van Eijk, Gerrit Koen, Dasja Pajkrt, Adithya Sridhar, Katja C. Wolthers

**Affiliations:** ^1^ OrganoVIR Labs, Department of Medical Microbiology, Amsterdam University Medical Centers (UMC), location Academic Medical Center, Amsterdam Institute for Infection and Immunity, University of Amsterdam, Amsterdam, Netherlands; ^2^ Emma Children’s Hospital Department of Pediatrics Infectious Diseases, Amsterdam University Medical Centers (UMC), location Academic Medical Center, University of Amsterdam, Amsterdam, Netherlands

**Keywords:** parechovirus, PeV-A, human organoids, enteroids, polarized epithelium, Transwell

## Abstract

Human parechovirus (PeV-A), one of the species within the *Picornaviridae* family, is known to cause disease in humans. The most commonly detected genotypes are PeV-A1, associated with mild gastrointestinal disease in young children, and PeV-A3, linked to severe disease with neurological symptoms in neonates. As PeV-A are detectable in stool and nasopharyngeal samples, entry is speculated to occur *via* the respiratory and gastro-intestinal routes. In this study, we characterized PeV-A1 and PeV-A3 replication and tropism in the intestinal epithelium using a primary 2D model based on human fetal enteroids. This model was permissive to infection with lab-adapted strains and clinical isolates of PeV-A1, but for PeV-A3, infection could only be established with clinical isolates. Replication was highest with infection established from the basolateral side with apical shedding for both genotypes. Compared to PeV-A1, replication kinetics of PeV-A3 were slower. Interestingly, there was a difference in cell tropism with PeV-A1 infecting both Paneth cells and enterocytes, while PeV-A3 infected mainly goblet cells. This difference in cell tropism may explain the difference in replication kinetics and associated disease in humans.

## 1 Introduction

*Parechovirus* (PeV) is a genus within the *Picornaviridae* family that is composed of positive sense single-stranded RNA viruses. This genus is divided into six different species of which species A (PeV-A) causes disease in humans, especially below the age of 1 year (de Crom et al., 2016; Sridhar et al., 2019). PeV-A is subdivided into 19 genotypes, of which PeV-A1 and PeV-A3 are the most prevalent genotypes (Brouwer et al., 2019).

PeV-A are highly prevalent viruses globally, with a seroprevalence as high as 100% in adults (Tanaka et al., 2016; Brouwer et al., 2019; Karelehto et al., 2019). PeV-A primarily infect young children while their prevalence in adults is very low (Brouwer et al., 2020). The most common illnesses due to PeV-A infection are gastrointestinal and respiratory disease. However, central nervous system (CNS) disease is also observed with cases of sepsis, meningitis, and encephalitis (Romero and Selvarangan, 2011). The severe CNS pathology is commonly associated with PeV-A3 genotype, but no clear explanation for this relation has been found (Benschop et al., 2006; Harvala et al., 2010; de Crom et al., 2016). Despite their clinical relevance, these viruses have been understudied and therapy is not available. Due to the detection of PeV-A primarily in stool and nasopharyngeal samples, transmission is hypothesized to occur *via* the fecal–oral or respiratory routes similar to enteroviruses (Romero and Selvarangan, 2011; Sridhar et al., 2019).

As PeV-A1 and PeV-A3 are associated with different disease symptoms, we hypothesized that PeV-A1 and PeV-A3 may differ in tropism and infection routes (Westerhuis et al., 2013). We previously reported on infection of the human airway epithelium (HAE) model, where both PeV-A1 and PeV-A3 showed a similar entry and cell tropism. However, PeV-A3 induced a stronger immune response in these airway cultures as compared to PeV-A1. Moreover, viral replication was observed preferentially upon basolateral infection, suggesting that the airway may be a secondary site of infection rather than a primary site (Karelehto et al., 2018). To further characterize this hypothesis on infection routes and primary entry site, route of infection of the gastrointestinal tract should also be studied.

**Figure 1:**
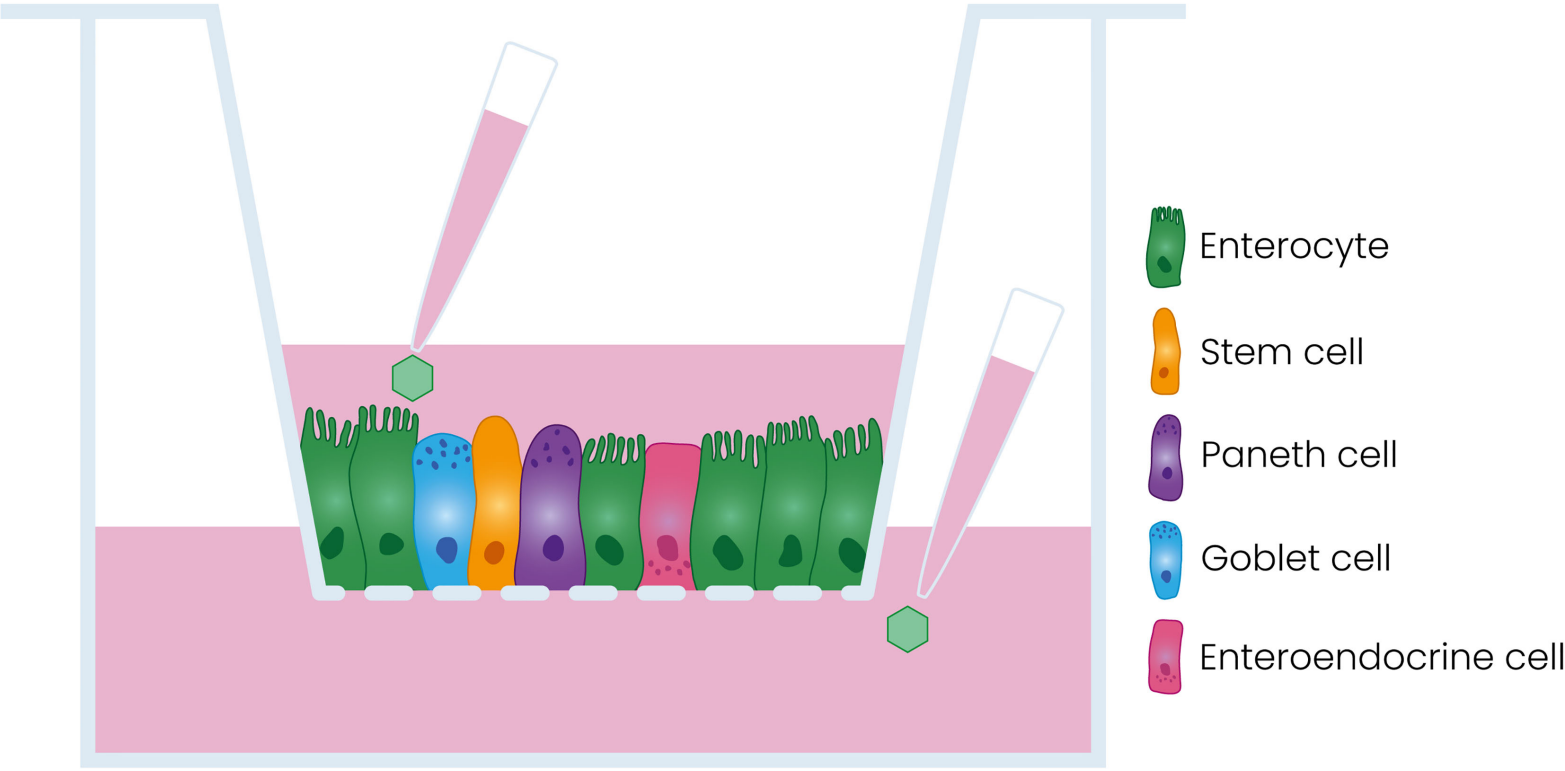
Schematic representation of the model used in this study. Dissociated enteroids are seeded on a porous Transwell**^®^** insert where they form a polarized monolayer containing the different cell types of the intestinal epithelium.

Human enteroids are three-dimensional (3D) structures composed of intestinal epithelial cells including enterocytes, Paneth cells, goblet cells, enteroendocrine cells, and stem cells. Enteroids are derived from primary intestinal stem cells and can be cultured long-term (Sato et al., 2009). Enteroids are excellent models to study pathogen interactions with the human intestinal epithelium (Drummond et al., 2016; Ettayebi et al., 2016; Saxena et al., 2016; García-Rodríguez et al., 2020). For viral studies, enteroids have been adapted to enhance their utility. One of these adaptations involves the generation of a two-dimensional (2D) monolayer on a Transwell^®^ insert, resulting in a polarized monolayer consisting of the different cell types found in the intestinal epithelium and access to both the apical and the basolateral side of the epithelium (**Figure 1**) (Noel et al., 2017; Good et al., 2019; Roodsant et al., 2020).

In this work, we use this fetal enteroid-derived model as a surrogate for the neonatal and infant gut to characterize PeV-A entry in the intestinal epithelium (Roodsant et al., 2020).

## 2 Materials and Methods

### 2.1 Cell Lines and Viruses

#### 2.1.1 Cell Lines

The following cell lines were used for virus culture: HT-29 cells (human colorectal adenocarcinoma; ATTC, Manassas, USA), LLCMK2 cells (rhesus monkey kidney cells, kindly provided by the Municipal Health Services, Rotterdam, the Netherlands), RD-99 (rhabdomyosarcoma, kindly provided by the National Institute of Public Health and the Environment, RIVM, Bilthoven, the Netherlands), and Vero (African green monkey kidney, kindly provided by RIVM, Bilthoven, the Netherlands). Cell lines were cultured in Eagle’s minimum essential medium (EMEM, Lonza, Basel, Switzerland) supplemented with 8% heat-inactivated fetal bovine serum (FBS, Sigma-Aldrich, St. Louis, USA), 100 U/ml each penicillin and streptomycin (Pen-Strep, Lonza, Basel, Switzerland), 1% (v/v) non-essential amino acids (100x, ScienceCell Research Laboratories, California, USA), and 0.1% L-glutamine (Lonza, Basel, Switzerland). All cell lines were incubated at 37°C, 5% CO_2_ and passaged every 7 days.

#### 2.1.2 Viruses

The PeV-A1 Harris strain was obtained from the RIVM (Bilthoven, the Netherlands) and cultured on HT-29 cells. The PeV-A3 152037 strain, a Dutch isolate from 2001 adapted to cell culture, was cultured on LLCMK2 cells. PeV-A3 A308-99 was a kind gift from Dr. Shimizu, National Institute of Infectious Diseases, Tokyo, Japan, and was cultured on HT-29 cells. Clinical isolates from stool specimens were cultured for one passage on a specific cell line and used afterwards for infections in enteroids, see **Table 1**.

**Table 1:** Clinical isolates used in the study.

Reference	Genotype	Year of collection	Patient age (years)	Patient sex	Clinical material	Cell line
52967	PeV-A1	2009	<1	Female	Stool	HT-29
51067	PeV-A1	2010	<1	Male	Stool	HT-29
51825	PeV-A3	2010	<1	Male	Stool	RD-99
51903	PeV-A3	2012	<1	Male	Stool	Vero

### 2.2 Enteroid Culture

Fetal enteroids were generated as described previously (Roodsant et al., 2020). Briefly, human fetal small intestine was cut open longitudinally and divided into small pieces ~5 mm each. The pieces were washed thoroughly with cold phosphate saline buffer (PBS, Lonza, Basel, Switzerland). Subsequently, the tissue was incubated in 2 mM ethylenediaminetetraacetic acid (EDTA, Avantor, Radnor, USA) in PBS for 30 min at 4°C on a roller. After incubation, the tissue was washed in 10% (v/v) FBS in PBS and the supernatant containing the intestinal crypts was collected and passed through a cell strainer (70 µm). The resulting crypts were suspended in Matrigel^®^ (Corning, New York, USA) and plated in three 10-µl droplets in a 24-well plate. The resulting domes were incubated at 37°C for 5 min to allow Matrigel^®^ polymerization and subsequently covered with 500 µl of IntestiCult™ Organoid Growth Medium (STEMCELL™ Technologies, Cambridge, UK) containing 100 U/ml Pen-Strep. Cultures were incubated at 37°C, 5% CO_2_, and full medium changes were performed every 2–3 days. Every 5–7 days, enteroids were passaged by mechanical dissociation.

### 2.3 Enteroid Monolayer Culture

Monolayer cultures were established as described previously (Roodsant et al., 2020). In short, Transwell^®^ inserts (HTS Transwell 24-well plate, Corning, New York, USA) were coated with 100 µl of 20 µg/ml collagen type I (rat tail, Ibidi, Gräfelfing, Germany) in 0.01% (v/v) acetic acid for 1 h at room temperature (RT), and washed thoroughly with PBS before cell seeding. Fetal enteroids were enzymatically dissociated into single cells using TrypLE™ (Gibco, Thermo Fisher Scientific, Waltham, USA) and diluted to 10^6^ cells/ml. One hundred microliters of this cell solution (10^5^ cells per insert) was seeded on the apical compartment of the Transwell^®^ and 600 µl of medium was added to the basolateral compartment. For the first 3 days, cells were cultured in IntestiCult™ with 10 µM Y-27632 (Sigma-Aldrich, St. Louis, USA), after which medium was changed to IntestiCult™. To promote cell differentiation from day 7 onwards, the medium used was a 1:1 mixture of the Basal Component of IntestiCult™ and Advanced Dulbecco’s Modified Eagle Medium/Nutrient Mixture F-12 (DMEM/F12, Gibco, Thermo Fisher Scientific, Waltham, USA) supplemented with 100 U/ml Pen-Strep, 7.5 mM HEPES (Sigma-Aldrich, St. Louis, USA) and 0.5× Glutamax (Thermo Fisher Scientific, Waltham, USA). Medium was refreshed every 2–3 days.

To monitor monolayer formation, trans-epithelial electrical resistance (TEER) was measured at days 3, 7, 11, and 14 post seeding with a EVOM-2 voltohmmeter (World Precision Instruments, Sarasota, USA). Resistance values were corrected using an empty Transwell^®^ and multiplied by the surface area of the insert to obtain the TEER (Ω × cm^2^). Only inserts with TEER ≥ 300 Ω × cm^2^ were used for the infections.

### 2.4 Monolayer Infection

Transwell^®^ cultures from crypts isolated from fetal donors of 18 weeks, 19 weeks, and 20 weeks were infected using technical duplicates per donor with the different viral strains. For the lab-adapted strains, cultures were infected with 10^5^ 50% tissue culture infectious dose (TCID50) (≈ 0.1 multiplicity of infection, MOI) of the specific virus in 50 µl of medium in either the apical or the basolateral compartment. For basolateral infection, 50 µl of the viral inoculum was added to the basolateral medium. For the clinical isolates, ~10^6^ viral copies in 50 µl were used to infect each insert. Monolayers were incubated for 2 h at 37°C with 5% CO_2_ and subsequently washed three times with PBS, both in the apical and the basolateral compartment. Medium was replenished in both compartments and the inserts were incubated for 10 min, after which the 0-h time point was collected, removing 100 µl from the apical and the basolateral side. Subsequently, the medium was replenished and collection at 1, 2, 3, and 4 days post infection (dpi) was performed.

### 2.5 PeV-A Detection by RT-qPCR

Viral RNA was isolated from 25 µl of the apical and basolateral samples using the Bioline Isolate II RNA mini kit (Meidian Bioscience^®^, Cincinatti, USA) according to the manufacturer’s instructions. Forty microliters of the eluted RNA was reverse-transcribed and 5 µl of the cDNA was used for real-time quantitative PCR (RT-qPCR) targeting the 5’ untranslated region of the PeV-A genome (Benschop et al., 2008) (see primer sequence in **Supplementary Table S2**). qPCR was performed on a CFX Connect Real-Time PCR Detection System (Bio-Rad, California, USA), and C_q_ values were transformed into viral copies using a standard curve with known concentrations of the viral genome.

### 2.6 PeV-A Detection by TCID50

Samples that showed the highest copy number were also analyzed by TCID50 and compared to the 0-h time point to determine the number of infectious particles using the Reed and Muench method (Reed and Muench, 1938). PeV-A1 Harris, PeV-A3 A308-99, PeV-A1 52967, and PeV-A1 51067 were titrated in HT-29 cells; PeV-A3 152037 was titrated in LLCMK2 cells; PeV-A3 51825 was titrated in RD-99 cells; and PeV-A3 51903 was titrated in Vero cells.

### 2.7 Immunofluorescence

Infected monolayers with the different PeV-A1 and PeV-A3 strains were fixed 7 dpi with 4% (v/v) formaldehyde (Sigma-Aldrich, St. Louis, USA) in PBS for 15 min at RT. After fixation, inserts were washed three times with PBS and stored in PBS at 4°C until staining. Membranes with the intestinal monolayers were excised from the Transwell^®^ inserts and permeabilized in ice-cold methanol for 10 min at −20°C. Subsequently, methanol was washed with 0.5% (v/v) Tween^®^ 20 (Sigma-Aldrich, St. Louis, USA) in PBS and blocking was performed using BlockAid™ Blocking Solution (Thermo Fisher Scientific, Waltham, USA), for 1 h on a shaker. PeV-A staining was performed using rabbit hyperimmune serum (kindly provided by Dr. Susi, University of Turku, Finland) (Karelehto et al., 2018). Specific labeling for cell types of the intestinal epithelium was performed using the following primary antibodies: anti-human mouse mucin2 (MA5-12345, Thermo Fisher Scientific, Waltham, USA), anti-human mouse villin (SC-58897, Santa Cruz Biotechnology, Dallas, USA), and anti-human mouse lysozyme (MA5-13096, Thermo Fisher Scientific, Waltham, USA). Monolayers were incubated with the primary antibodies overnight at 4°C. After extensive washing with 0.5% (v/v) Tween20, secondary staining was performed with donkey anti-mouse ALEXA546 (A10036, Thermo Fisher Scientific, Waltham, USA) and anti-rabbit ALEXA647 (A-31573, Thermo Fisher Scientific, Waltham, USA). All antibodies were diluted in 3% (w/v) bovine serum albumin (BSA) in PBS; for specific dilutions, see **Supplementary Table S1**. Cultures were incubated with the secondary antibodies for 1 h at RT after which quenching was performed using ReadyProbes™ Tissue Autofluorescence Quenching Kit (R37630, Thermo Fisher Scientific, Waltham, USA) for 5 min. Quenching solution was removed and washed three times with 0.5% (v/v) Tween^®^ 20, and 3 µM DAPI (Thermo Fisher Scientific, Waltham, USA) was added for nuclei staining. Finally, after washing the DAPI, inserts were mounted using ProLong™ Glass Antifade Mountant (P36980, Thermo Fisher Scientific, Waltham, USA). Slides were imaged using Leica TCS SP8-X microscope with HC Plan Apochromat 63× oil objective and Leica LAS AF Software (Leica Microsystems, Wetzlar, Germany). Z-stacks were taken, and 3D reconstructions were made using the LAS-X 3D software.

### 2.8 Statistical Analysis

For the replication kinetics, the relative increase in RNA copies was calculated dividing the RNA copies of a specific time point by the average RNA copies at the 0-h time point. The mean value of the technical replicates (*n* = 2) and biological replicates (*n* = 3) for each time point and apical and basolateral inoculation was compared within each virus against the 0-h time point using a non-parametric one-way ANOVA with multiple comparisons using GraphPad Prism 8 (GraphPad Software Inc., La Jolla, USA). Differences were considered significant when the *p*-value was <0.05. To determine differences between apical and basolateral infection, the last time point of apical and basolateral inoculation was compared with a Mann–Whitney test using the same software.

## 3 Results

### 3.1 Parechovirus A Laboratory-Adapted Genotype 1, but Not 3, Replicates Well in the Intestinal Epithelium

To characterize PeV-A infection of the intestinal epithelium, we inoculated enteroid-derived monolayers with lab-adapted strains of PeV-A1 and PeV-A3 that are frequently used for viral tropism studies (Hyypiä et al., 1992; Ito et al., 2004; Benschop et al., 2006; Westerhuis et al., 2013). For PeV-A1 infection, the PeV-A1 Harris strain was used, and for PeV-A3, the two lab-adapted strains PeV-A3 152037 and A308-99 were used.

**Figure 2:**
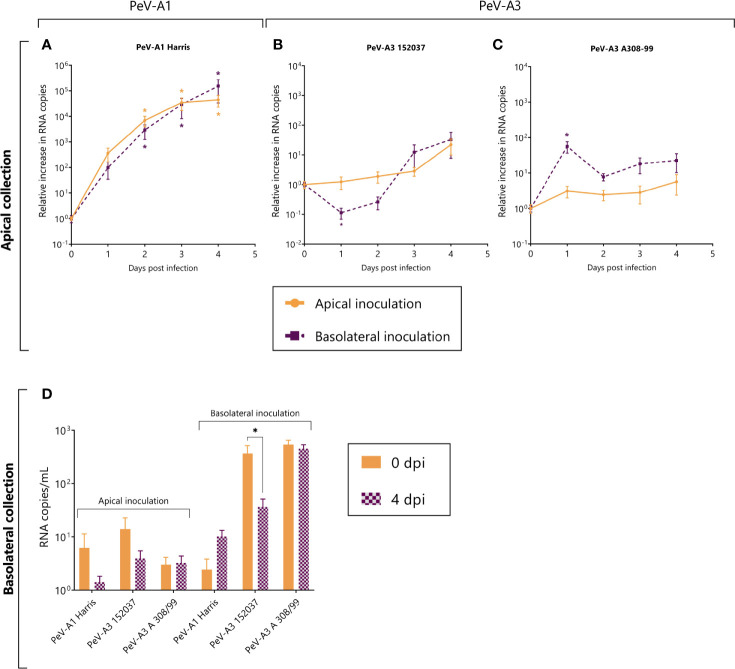
Replication kinetics of lab-adapted strain PeV-A1 (Harris) and two lab-adapted PeV-A3 strains (PeV-A3 152037 and A308-99) in intestinal monolayers. **(A–C)** Represents the relative increase in RNA copies in samples collected from the apical compartment after both apical and basolateral inoculation. **(D)** Represents the total amount of viral copies at 0 dpi and 4 dpi in the basolateral compartment, after either apical or basolateral inoculation. In all cases, data represent the mean ± SEM of two technical replicates in three biological replicates. * *p*-value < 0.05. dpi = days post infection.

Replication was observed for the PeV-A1 Harris strain (**Figure 2A**) while no replication or low levels of replication were observed for both of the PeV-A3 lab-adapted strains (**Figures 2B, C**). For PeV-A1 Harris, replication occurred after both apical and basolateral inoculation with shedding at the apical compartment of the model. There was no significant difference in replication level between apical and basolateral inoculation (**Figure 2A**). For PeV-A3 152037 and A308-99, we could not detect any significant increase in RNA copies over time (**Figure 2B**). For PeV-A3 A308-99, a slight increase was observed at 24 h after basolateral inoculation, but the RNA levels were reduced after that time point, indicating that replication did not occur (**Figure 2C**).

In terms of shedding to the basolateral compartment, we did not observe any significant increase in RNA copies after 4 days for any of the strains, indicating that these viruses are not being shed into the basolateral side of the intestinal epithelium model (**Figure 2D**).

**Figure 3:**
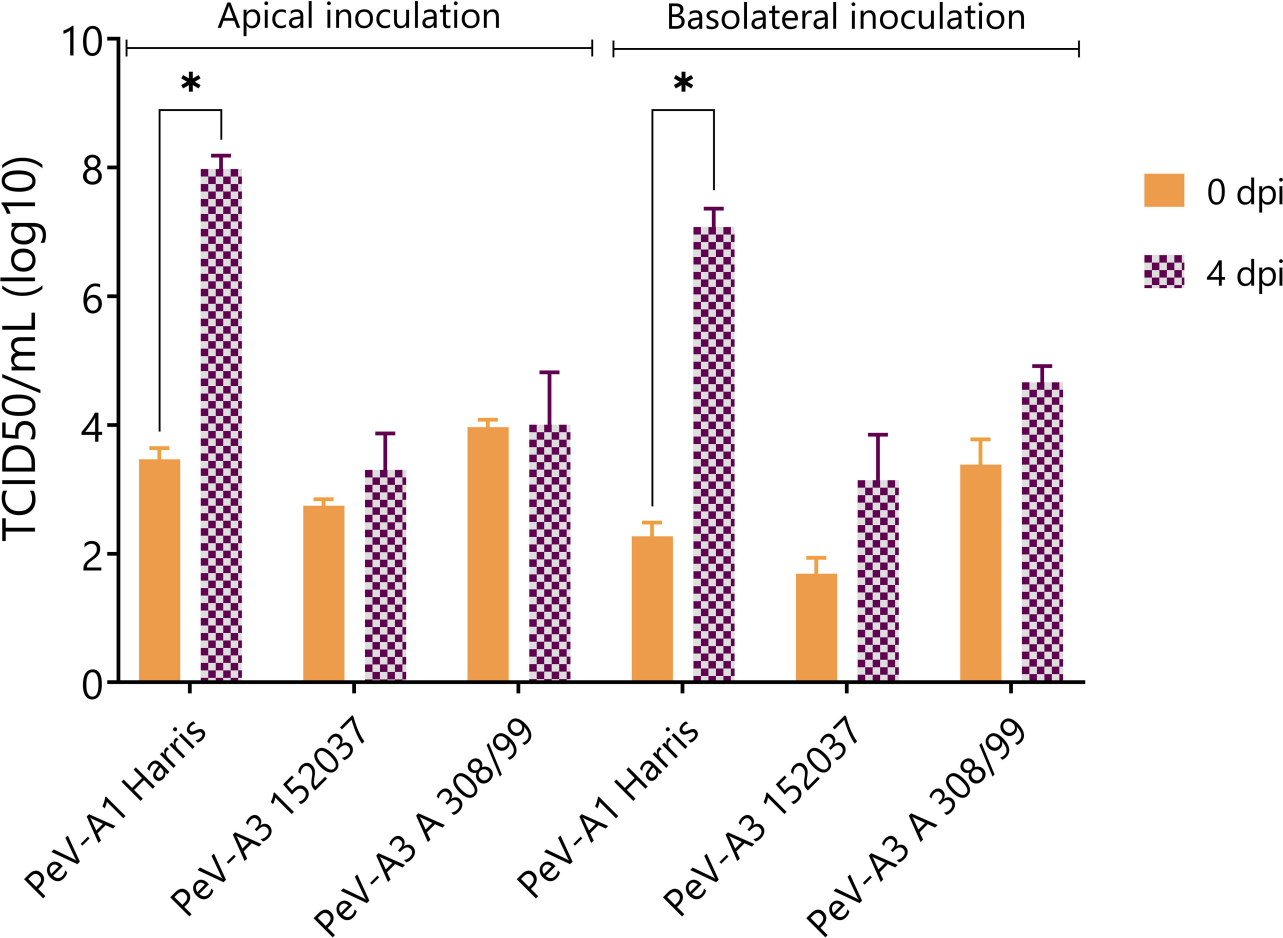
Virus titer at 0 and 4 dpi of apically collected samples. Titers were determined by TCID50. In all cases, data represent the mean ± SEM of two technical replicates in three biological replicates. * *p*-value < 0.05. dpi = days post infection.

In order to confirm the generation of infectious viral particles, TCID50 on permissive cell lines was performed and the titers at time points 0 and 4 days after infection were compared. Since no shedding was observed in the basolateral compartment, only apically collected samples were taken into account. In line with the qPCR data, PeV-A1 Harris showed a significant increase in viral titers both after apical and basolateral inoculation. Even though there was an increase in viral titers for the two lab-adapted strains of PeV-A3, this increase was not significant (**Figure 3**).

### 3.2 PeV-A3 Clinical Isolates Replicate in the Intestinal Epithelium, but to a Lesser Extent Than PeV-A1

**Figure 4:**
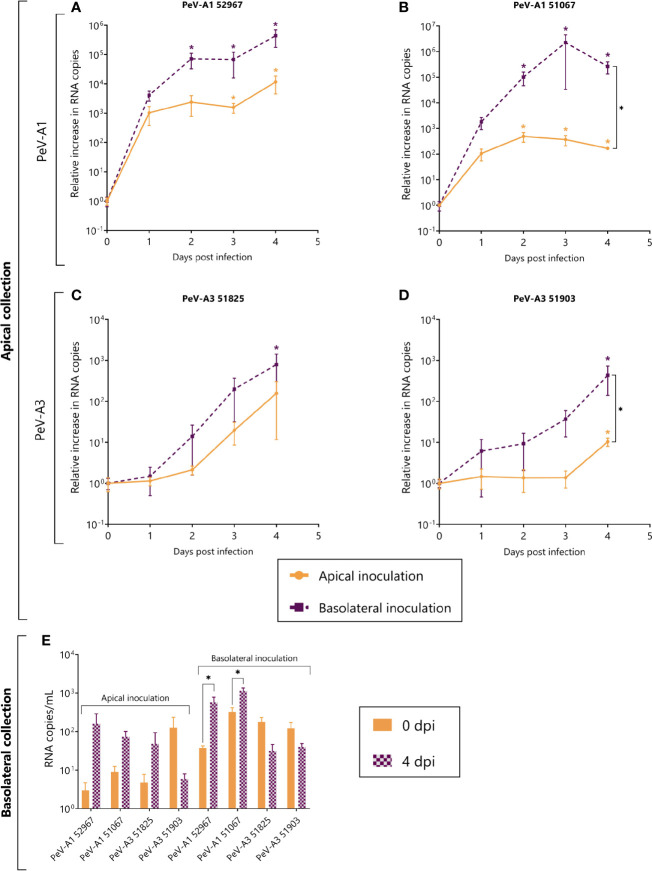
Replication kinetics of clinical isolates of PeV-A1 and PeV-A3 in intestinal monolayers. **(A–D)** Represent the relative increase in RNA copies in samples collected from the apical compartment after both apical and basolateral inoculation; **(A, B)** are PeV-A1 clinical isolates from stool samples; **(C, D)** are PeV-A3 clinical isolates from stool samples. **(E)** Represents the total amount of viral copies at 0 dpi and 4 dpi in the basolateral compartment, after either apical or basolateral inoculation. In all cases, data represent the mean ± SEM of two technical replicates in three biological replicates. * *p*-value < 0.05. dpi = days post infection.

Due to the lack of replication of both PeV-A3 laboratory-adapted strains, we further characterized replication in the intestinal epithelium using clinical isolates obtained from a clinical sample and passaged only once on a cell line, thereby allowing the original virus population little to no adaptation. Replication was observed for both clinical PeV-A1 (**Figures 4A, B**) and PeV-A3 isolates. PeV-A3 clinical isolates showed replication albeit to a lower extent as compared to the clinical PeV-A1 isolates (**Figures 4C, D**). Contrary to what was observed for the lab-adapted strain, the clinical PeV-A1 isolates showed enhanced replication after basolateral inoculation; this difference was significant for isolate 51067 (**Figure 4B**). Infection also occurred after apical inoculation, with increasing RNA copies in the apical compartment over time.

As compared to PeV-A1, PeV-A3 isolates replicated to a lesser extent and significant replication was only observed after basolateral inoculation with apical sampling (**Figures 4C, D**). Although a significant increase was observed after apical inoculation for isolate 51903 (**Figure 4D**), the relative increase was only ~10-fold. This indicates that replication for PeV-A3 clinical isolates in this model occurs mainly after basolateral inoculation with apical shedding.

Similarly to what was observed for the lab-adapted strains, after apical inoculation, the amount of RNA copies did not increase in the basolateral compartment (**Figure 4E**). However, when analyzing basolateral shedding after basolateral inoculation, there was a significant increase in viral copies of the two clinical isolates of PeV-A1 (**Figure 4E**). The apparent increase in viral copies of the clinical isolates of PeV-A1 can also be occasioned by viral particles leaking from the apical compartment after the monolayer loses its integrity due to cell damage (**Supplementary Figures S1, S2**).

**Figure 5:**
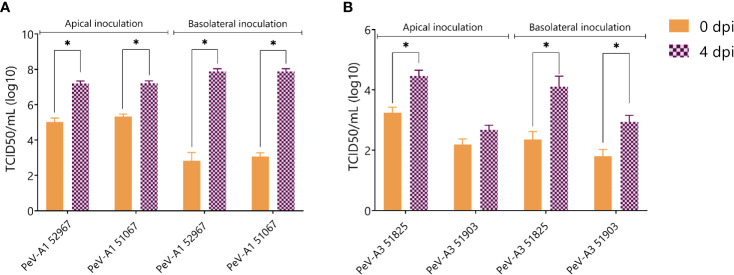
Virus titer at 0 and 4 days post infection of apically collected samples for **(A)** PeV-A1 clinical isolates and **(B)** PeV-A3 clinical isolates. Titers were determined by TCID50. In all cases, data represent the mean ± SEM of two technical replicates in three biological replicates. * *p*-value < 0.05. dpi = days post infection.

The generation of new infectious particles was confirmed with TCID50. For both PeV-A1 clinical isolates, there was a significant increase in infectious particles by day 4, after both apical and basolateral infection (**Figure 5A**). For the PeV-A3 isolates, the development of a cytopathic effect (CPE) in the cell lines was slower than for PeV-A1, taking up to 2 weeks for the first positive CPE wells. This slower CPE formation was probably due to the specific isolates not being adapted to growth on cell lines. However, 3 weeks after the inoculation, a clear CPE was observed with a significant increase in TCID50 for both PeV-A3 clinical isolates after basolateral inoculation. For PeV-A3 51825, we also observed a significant increase in TCID50 after apical inoculation (**Figure 5B**).

### 3.3 Different Cell Tropism for PeV-A1 and PeV-A3

**Figure 6:**
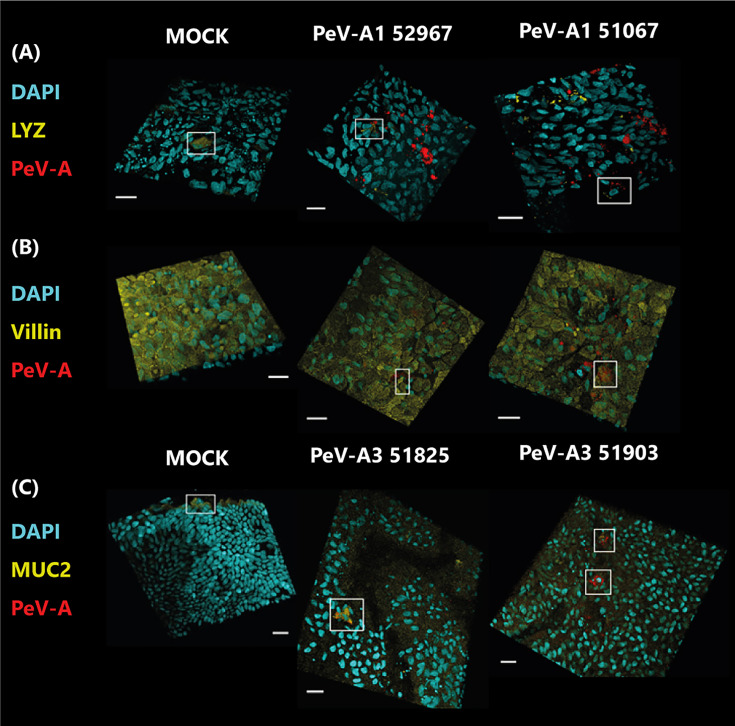
Confocal images of infected cultures with clinical isolates of PeV-A1 and PeV-A3. In panel **(A)**, cultures were stained with Paneth cell marker (LYZ) in yellow and PeV-A antibody in red; in panel **(B)**, they were stained with enterocyte marker (villin) in yellow and PeV-A antibody in red, and in panel **(C)**, cultures were stained with goblet cell marker (MUC2) in yellow and PeV-A antibody in red. In all cases, nuclei were stained with DAPI in cyan. Scale bars in white represent 20 µm, and the boxes indicate positively stained cells for the cell marker in the MOCK cases and for viral and cell marker staining in the infected images.

To determine if the lower replication levels of PeV-A3 could be explained by differential tropism and therefore by a difference in the amount of target cells present, viral tropism was studied by immunolabeling with PeV-A antibody and specific antibodies for the Paneth cell marker lysozyme (**Figure 6A**), the enterocyte cell marker villin (**Figure 6B**), and the goblet cell marker mucin-2 (**Figure 6C**).

For PeV-A1 Harris, only a few infected cells could be observed, and for the lab-adapted PeV-A3 152037, no positive virus staining could be found (data not shown). However, different cell tropism was observed for the clinical isolates of PeV-A1 and PeV-A3. Cultures infected with PeV-A1 clinical isolates showed infected cells positive for the Paneth cell marker lysozyme; however, not all infected cells were positive for this marker (**Figure 6A**), indicating that both isolates were able to infect Paneth cells, but also other cell type(s). These PeV-A1 strains were also able to infect cells that were positive for the enterocyte marker, villin (**Figure 6B**). While no colocalization with the goblet cell marker (MUC2) was observed for any of the PeV-A1 clinical isolates (**Supplementary Figure S3**). These data indicate that PeV-A1 clinical isolates are able to infect Paneth cells and enterocytes.

Clinical isolates of PeV-A3 showed PeV-A-infected cells that were positive for the goblet cell marker mucin-2, and for isolate 51903, infected cells negative for this marker were also observed (**Figure 6C**). However, no colocalization with other markers was observed (data not shown). In conclusion, PeV-A3 clinical isolates are able to infect mainly goblet cells and as yet unidentified cells.

## 4 Discussion

In this study, we showed susceptibility of the fetal intestinal epithelial model to PeV-A1 and PeV-A3 infection. For both genotypes, replication was more efficient when inoculation was performed from the basolateral side with subsequent viral release from the apical side. While the polarity of infection for PeV-A1 and PeV-A3 was similar, we identified different cell targets. PeV-A1 infected both Paneth cells and enterocytes. In contrast, PeV-A3 targeted mainly goblet cells.

Although PeV-A1 and PeV-A3 showed enhanced replication after basolateral inoculation, all PeV-A1 strains were also able to replicate after apical inoculation. We were not able to observe any replication of PeV-A3 after apical inoculation. However, similar to the closely related enteroviruses, entry is supposed to be *via* the gastrointestinal and/or the respiratory tract (Romero and Selvarangan, 2011; Sridhar et al., 2019). Indeed, infection of some common gastrointestinal viruses usually occurs from the apical part of the epithelium, as shown for human norovirus (HuNoV) and for enterovirus A71, another member of the *Picornaviridae* family. In contrast, for echovirus 11, a similar pattern of basolateral infection as in PeV-A3 infection was previously described (Good et al., 2019). For some virus types, the mechanisms of entry might require interaction with non-epithelial cells that are not present in the model, such as cells of the lymphoid tissue. These specialized regions of the intestine are called Peyer´s patches and contain a specific subset of cells called microfold (M) cells. M cells play a role in microbial protection with a capacity to sample antigens (Pfeiffer, 2010). Poliovirus, another member of the *Picornaviridae* family, has been hypothesized to enter the intestine through M cells (Pfeiffer, 2010). Moreover, when included in an enteroid-based monolayer, M cells were able to internalize and transport reovirus (double-stranded RNA viruses) across the epithelium (Ding et al., 2020), highlighting the importance of M cells in mucosal pathogen translocation. Another subset of cells that are not included in the model are mucosal immune cells. Co-cultures of enteroids with innate immune cells such as macrophages have already been established (Staab et al., 2020), and the role of these macrophages on sensing and transporting bacteria across the epithelium was demonstrated (Noel et al., 2017). We hypothesize that some of these cells, either resident immune cells or M cells, may play a role on PeV-A infection of the intestinal epithelium by transporting the virus to the basolateral part of the tissue, but further research is needed.

Although PeV-A3 is more pathogenic than PeV-A1 in neonates, the clinical PeV-A3 isolates used in our study showed lower replication efficiency than the PeV-A1 clinical strains. The difference in cell tropism found between genotypes could be one of the explanations for this. Goblet cells (the cell target for PeV-A3) represent approximately 10% of the cells in the intestinal epithelium (Karam, 1999) and also their presence in our model is quite low (Roodsant et al., 2020), while enterocytes (one of the cell targets of PeV-A1) account for >80% of the cells in the epithelium (Cheng and Leblond, 1974). Differences in cell tropism have also been described for adenovirus, with some types exclusively targeting goblet cells, while others are able to infect multiple cell types (Holly and Smith, 2018a). Similar to PeV-A3 cell tropism, other viruses like enterovirus A71 (Good et al., 2019) and human astrovirus can also infect goblet cells (Kolawole et al., 2019). For murine astrovirus, mucus production was increased upon infection in an animal model, and it was shown that this could help virus exit and/or dissemination to secondary sites of infection (Cortez et al., 2020; Cortez and Schultz-Cherry, 2021). Whether enhanced dissemination after goblet cell infection may play a role in the pathogenesis of PeV-A3 remains to be elucidated. In terms of cell tropism for PeV-A1, several viruses have been described to infect enterocytes, like human rotavirus (Saxena et al., 2016) or severe acute respiratory syndrome-coronavirus 2 (SARS-CoV-2) (Lamers et al., 2020). Conversely, the other cell target for PeV-A1, Paneth cells, has not been described as a cell target for many viruses; only mouse adenovirus 2 is known to infect Paneth cells (Holly and Smith, 2018b). Moreover, Paneth cells are present in a much lower percentage (<6%) in the intestinal epithelium (Cheng and Leblond, 1974), so they will probably have a lesser role in replication.

Next to a different cell tropism, technical issues such as accurate determination of virus input and output in organoid-based models, or donor variation in supporting infection, might play a role in the observed difference in replication levels. In addition, previous work from our group and others (Karelehto et al., 2018; Good et al., 2019) showed differences in eliciting an innate immune response between different virus genotypes. In the case of HuNoV, knockout of genes in human enteroids involved in the interferon pathways increased the replication of strain GII.3 HuNoV, but not of strain GII.4 (Lin et al., 2020). Similarly, within the *Enterovirus* genus, enterovirus A71 and echovirus 11 preferentially induced a specific immune response in human enteroids that lead to different replication kinetics (Good et al., 2019). In the case of PeV-A, after infection of a HAE model, PeV-A3 infection elicited an upregulation of genes involved in immune responses and inflammation, while this response was less acute for PeV-A1 (Karelehto et al., 2018). A different innate immune response may be elicited upon infection of our model with both genotypes, which could also explain the replication kinetics.

Another important finding of our study was the significant difference in replication between lab-adapted strains and clinical isolates. The lab-adapted strains of PeV-A3 were not able to infect the human enteroid culture model. It is well known that, due to their high mutation rate, RNA viruses are capable of adapting to a specific cell line after just a few passages (Sridhar et al., 2020). Some of these mutations may enhance viral entry by allowing binding to new receptors (Cagno et al., 2019) or promoting better attachment (Bochkov et al., 2016). Although some of these adaptations have been found *in vivo* after viral dissemination to secondary targets (Tseligka et al., 2018), most of them are never found in clinical isolates and are thought to be derived purely from their culture on cell lines (Vlasak et al., 2005; Cagno et al., 2019). It has also been shown that some mutations acquired during cell line culturing hamper viral growth *in vivo* or in primary cultures. Some examples include human parainfluenza for which an advantage acquired during cell culture made the virus noninfectious on HAE cultures (Palermo et al., 2009). Marburg virus also showed attenuation after serial cell culture passage and subsequent culture in a macaque model (Alfson et al., 2018), or even more recently SARS-CoV-2 for which culture in non-airway cell lines generated a mutation that reduced attachment on airway cell lines and airway organoids (Lamers et al., 2021). Based on these previous findings, we hypothesize that a similar pattern of mutations occurred in the lab-adapted strains of PeV-A3, hampering their growth on the primary cultures. Virus genome sequencing can confirm this hypothesis.

In conclusion, we showed that viral infection of PeV-A in our intestinal epithelium model is polarized with a preference for basolateral infection and that replication and cell tropism are genotype dependent. We also showed the difference between replication of clinical isolates and lab-adapted strains, which points out the need to use human-based models to study viral infection, as these models mimic the cell heterogeneity and complexity observed *in vivo*.

## Data Availability Statement

The raw data supporting the conclusions of this article will be made available by the authors, upon request, without undue reservation.

## Ethics Statement

Tissues were obtained with approval of the ethical committee of the Amsterdam UMC, together with approval of the experimental procedures by the HIS Mouse Facility (Amsterdam UMC). All methods were performed in accordance with the relevant guidelines and regulations, as stated in the Amsterdam UMC Research Code, in a certified laboratory (ISO15189 accreditation M304). The patients/participants provided their written informed consent to participate in this study.

## Author Contributions

Conceptualization: IG-R, AS, DP, and KW. Data curation: IG-R, HE, and GK. Formal analysis: IG-R. Funding acquisition: AS, DP, and KW. Investigation: IG-R, HE, and GK. Methodology: IG-R, HE, GK, and AS. Project administration: IG-R, AS, DP, and KW. Resources: HE, GK, and AS. Supervision: AS, DP, and KW. Validation: IG-R. Visualization: IG-R. Writing of the original draft: IG-R and AS. Writing, review and editing: IG-R, AS, DP, and KW. As described by CRediT [CRediT—Contributor Roles Taxonomy (casrai.org)]. All authors contributed to the article and approved the submitted version.

## Funding

This research was co-funded by the European Union’s Horizon 2020 Research and Innovation Programme under the Marie Sklowdowska-Curie Grant Agreement OrganoVIR (grant 812673) and the PPP Allowance (Focus-on-Virus) made available by Health Holland, Top Sector Life Sciences & Health, to the Amsterdam UMC, location Amsterdam Medical Center to stimulate public–private partnerships.

## Conflict of Interest

The authors declare that the research was conducted in the absence of any commercial or financial relationships that could be construed as a potential conflict of interest.

## Publisher’s Note

All claims expressed in this article are solely those of the authors and do not necessarily represent those of their affiliated organizations, or those of the publisher, the editors and the reviewers. Any product that may be evaluated in this article, or claim that may be made by its manufacturer, is not guaranteed or endorsed by the publisher.

## Abbreviations

2D, 2-dimensional; 3D, 3-dimensional; BSA, Bovine serum albumin; CNS, Central nervous system; CPE, Cytopathic effect; DMEM/F12, Dulbecco’s Modified Eagle Medium/Nutrient Mixture F-12; dpi, Days post infection; EDTA, Ethylenediaminetetraacetic acid; EMEM, Eagle´s minimum essential medium; FBS, Fetal bovine serum; HAE, Human airway epithelium; HuNoV, Human norovirus; LYZ, Lysozyme; M, Microfold; MOI, Multiplicity of infection; MUC-2, Mucin-2; PBS, Phosphate saline buffer; PeV, *Parechovirus*; PeV-A, *Parechovirus* species A; qPCR, Quantitative polymerase chain reaction; RIVM, National Institute of Public Health and the Environment; RT, Room temperature; SARS-CoV-2, Severe acute respiratory syndrome-coronavirus 2; TCID50, 50% tissue culture infectious dose; TEER, Trans-epithelial electrical resistance.
