# Supplementary material for: Parechovirus A Infection of the Intestinal Epithelium: Differences Between Genotypes A1 and A3
Source: Front Cell Infect Microbiol. 2021 Nov 1;11:740662. doi: 10.3389/fcimb.2021.740662 (PMC8591172; doi:10.3389/fcimb.2021.740662)
Supplement: Supplementary file 1 [file DataSheet_1.docx]

Supplementary Material

# Supplementary Figures and Tables

Supplementary Table 1. Primary antibody dilutions for immunofluorescence staining.

| **Antibody** | **Species** | **Target** | **Company** | **Catalogue number** | **Dilution** |
| --- | --- | --- | --- | --- | --- |
| Mucin 2 | Mouse | Goblet cells | Thermo Fisher Scientific | MA5-12345 | 1:100 |
| Villin | Mouse | Enterocytes | Santa Cruz Biotechnology | SC-58897 | 1:100 |
| Lysozyme | Mouse | Paneth cells | Thermo Fisher Scientific | MA5-13096 | 1:100 |
| PeV-A hyperimmne serum | Rabbit | PeV-A | -- | -- | 1:300 |

Supplementary Table 2. Primers and probe sequence for PeV-A viral RNA detection.

| **Primer** | **Sequence** |
| --- | --- |
| Forward | 5’-CTG GGG GCC AAA AGC CA-3’ |
| Reverse | 5’-GGT ACC TTC TGG GCA TCC TTC-3’ |
| Probe | (6’FAM)-5’-AAA CAC TAG TTG TAW GGC CC-3’ |


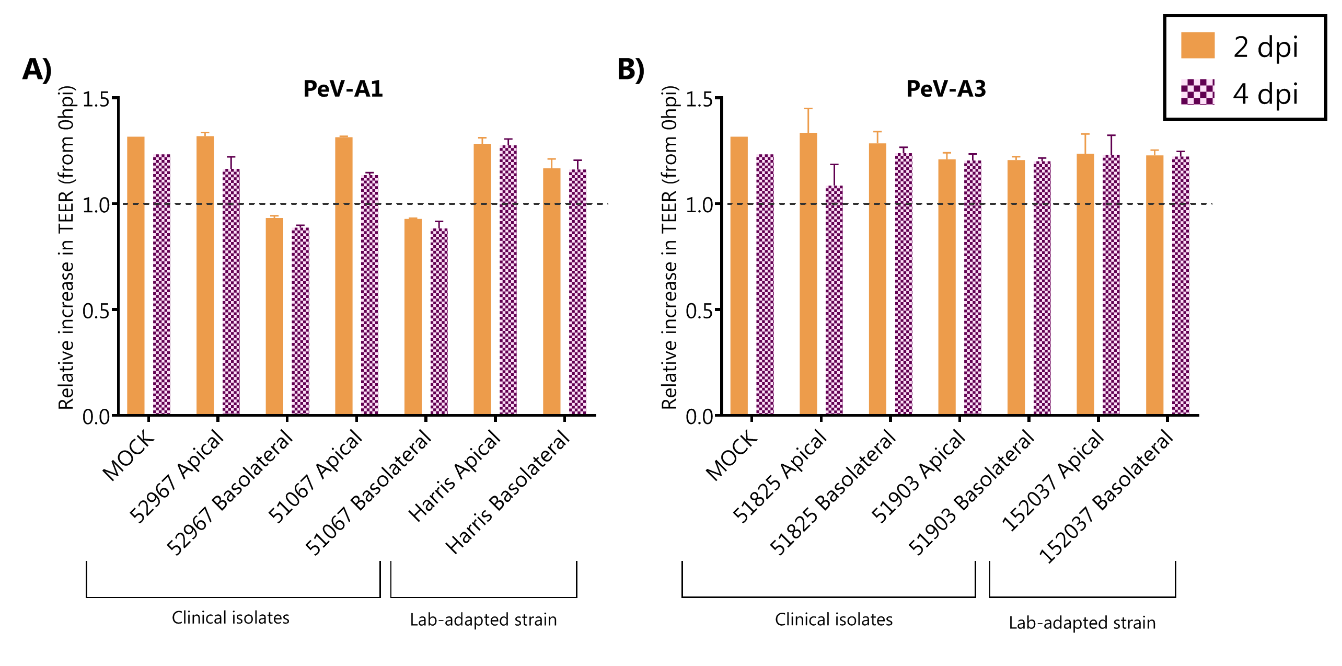


**Supplementary Figure 1.** Relative increase in trans-epithelial electrical resistance (TEER) values between the 0 dpi and 2 and 4 dpi. The dashed lines represent no change in TEER, values above the line indicate an increase in TEER and values underneath indicate a decrease. (A) Data for PeV-A1, (B) data for PeV-A3, both clinical isolates and lab-adapted strains. Data represents the mean ± SEM of 2 technical replicates in one donor.

| **MOCK**  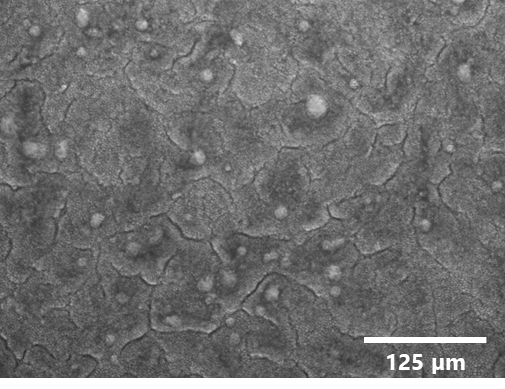 | |
| --- | --- |
| **PeV-A1 52967 Basolateral inoculation**  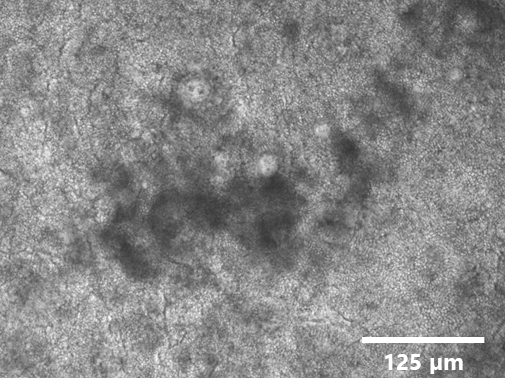 | **PeV-A1 51067 Basolateral inoculation**  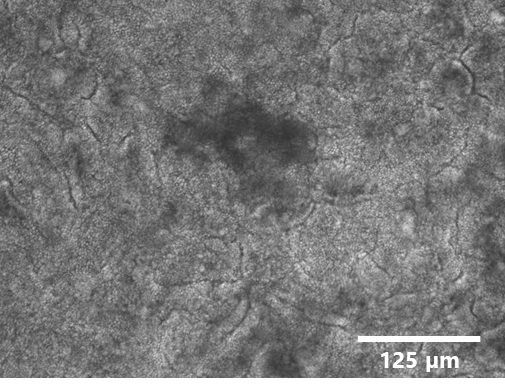 |
| **PeV-A3 51825 Basolateral inoculation**  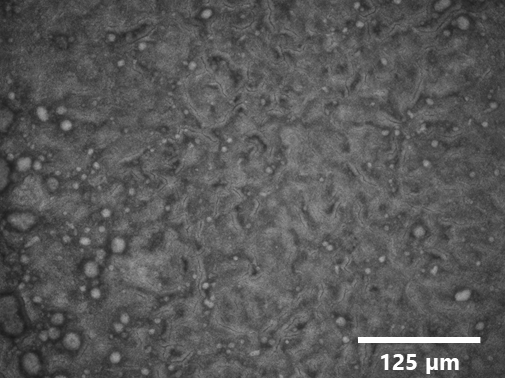 | **PeV-A3 51903 Basolateral inoculation**  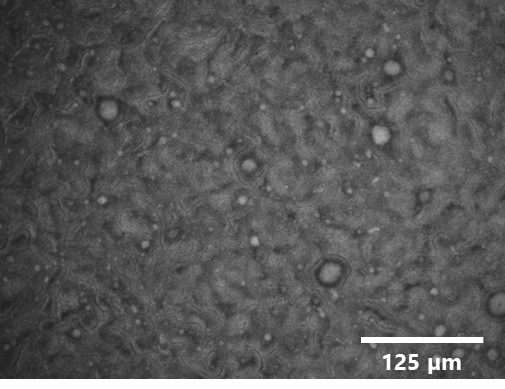 |

Supplementary Figure 2. Representative photos from the enteroid monolayers were taken 4 dpi with an EVOS microscope. CPE could not be observed for PeV-A3 infected monolayers.

|  | **PeV-A1 52967** | **PeV-A1 51067** |
| --- | --- | --- |
| **DAPI**  **MUC2**  **PeV-A** | **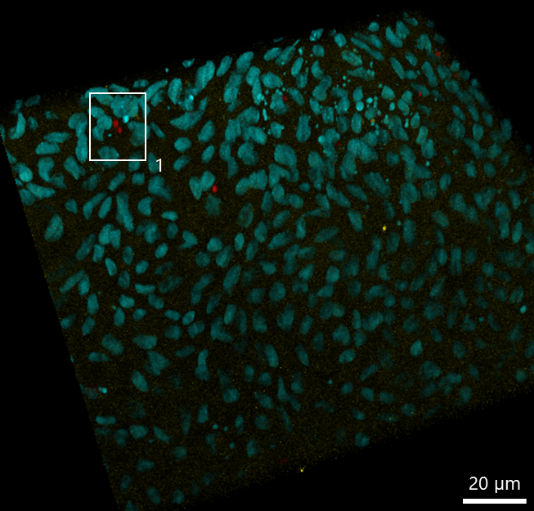** | **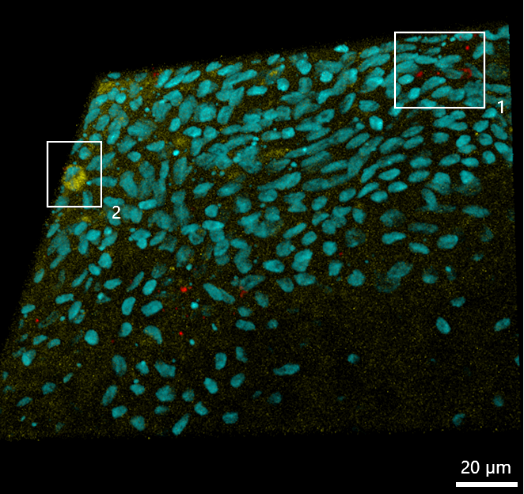** |

Supplementary Figure 3. Confocal images of infected cultures with clinical isolates of PeV-A1. Cultures were stained with goblet cell marker (MUC2) in yellow and PeV-A antibody in red. In all cases nuclei were stained with DAPI in cyan. Scale bars in white represent 20 µm, and the boxes indicate positively stained cells for the infected cells (1) and for the cellular marker (2). MOCK infected cultures can be found in the main text.
